# Universal scaling laws of collective human flow patterns in urban regions

**DOI:** 10.1038/s41598-020-77163-2

**Published:** 2020-12-08

**Authors:** Yohei Shida, Hideki Takayasu, Shlomo Havlin, Misako Takayasu

**Affiliations:** 1 Department of Mathematical and Computing Science, School of Computing, Tokyo Institute of Technology, Yokohama, 226-8502 Japan; 2 Sony Computer Science Laboratories, Tokyo, 141-0022 Japan; 3 Institute of Innovative Research, Tokyo Institute of Technology, Yokohama, 226-8502 Japan; 4 Department of Physics, Bar-Ilan University, Ramat-Gan, 52900 Israel; 5 Tokyo Tech World Research Hub Initiative (WRHI), Institute of Innovative Research, Tokyo Institute of Technology, Yokohama, 226-8502 Japan

**Keywords:** Nonlinear phenomena, Phase transitions and critical phenomena

## Abstract

Detail observation of human locations became available recently by the development of information technology such as mobile phones with GPS (Global Positioning System). We analyzed temporal changes of global human flow patterns in urban regions based on mobile phones’ GPS data in 9 large cities in Japan. By applying a new concept of drainage basins in analogous to river flow patterns, we discovered several universal scaling relations. These include, the number of moving people in a drainage basin of diameter *L* is proportional to $L^3$ in the morning rush hour, which is surprisingly different from reasonable intuition of proportionality to the 2 dimensional area, $L^2$. We show that this unexpected 3 dimensional feature is related to the strong attraction of the city center to become a 3 dimensional structure due skyscrapers.

## Introduction

Scientific studies of human location data have a long history starting from finding empirical laws of migration using population data in 1885^1^. Since then and until 2000 human location data collection was limited to questionnaires or population surveys over long time periods. In the twentieth century, flow of cars was extensively analyzed because human flow could not yet be tracked^2^. The situation has changed drastically in the beginning of this century by the modern information technology^3–6^. For example, mobile phones with GPS provide detailed information of locations of enormous number of people, simultaneously. By analyzing such detailed observational data, study of human mobility became much more precise and intensive. Recent studies of human mobility can be roughly categorized into two groups: One is focusing on statistical properties of *individual* trajectories^7–9^, and the other is global *migration* between cities^10,11^. Microscopically, trajectories of human locations may look random, but actually, they are very different from Brownian motion of fine particles. It is found that human trajectories can be approximated by the Levy flight model of power law length distribution of jumps^12^. Individual trajectories can be classified into several social activity classes^13–15^, and predictability of each trajectory has been discussed^16,17^. Beside daily activities, researchers also analyzed specific important phenomena such as panic behavior right after sever earthquakes^18,19^ , and resilient features of traffic congestions^20^. Macroscopically, in the field of human migration, the so called gravity law has been widely applied^21–23^. Besides the gravity model, the intervening opportunity class model, where the flow amount is proportional to the opportunity of the destination and inversely proportional to the intervention opportunity between the origin and the destination, has been widely studied^24–28^. Also, probabilistic human mobility prediction are widely performed for congestion and advertisement optimization^29–32^. Recently, the potential within the big cities has been estimated using the vector field generated from the Origin-Destination matrix, which includes the number of people traveling between all pairs of spots^33^. However, studies of collective human flow (vector field) within scale of cities, which we call here mesoscopic scales, have been rarely addressed.

In this paper, we introduce and develop a framework to perform a mesoscopic analysis of collective human mobility, within urban areas. We analyze GPS location data of mobile phones with the information of velocity and location, and observe the temporal evolution of collective flow patterns of human mobility within big cities. In our framework we regard human flow like water flow and observe temporal changes of drainage basin structures within and around large cities applying the concept of power laws.

## Results

### Discretized human flow patterns in a city

**Figure 1:**
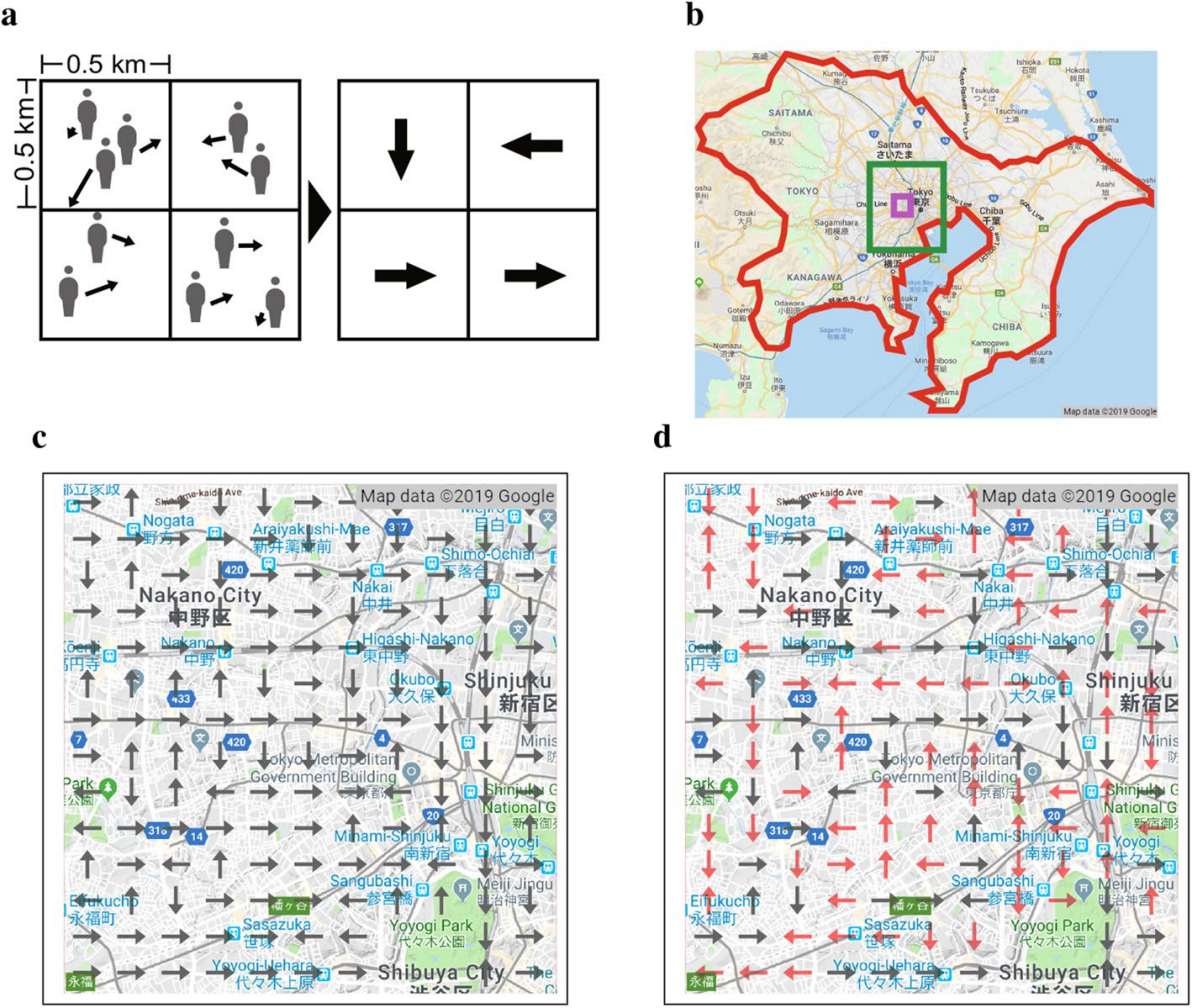
Velocity discretization and different flow patterns. **a** Schematic figures demonstrating the way we discretize the human flow. Left: The velocity of people in each square of $500\times 500 \hbox { m}^2$ is calculated by evaluating their average velocity components over those people who are moving in the square during the observation interval of 30 min. Right: We assign one of the four representative unweighted directions (north, east, south, west) choosing the dominant direction of the average velocity in each square. No direction is assigned for those squares that do not include moving people. **b** The area we analyze here is the greater Tokyo metropolitan. Tokyo and neighboring 3 prefectures are shown surrounded by red. The purple square shows the area of **c**,**d**. The green square shows the area covered by Fig. 2b,c shown below. **c**,**d** Discretized flow patterns on the map in the morning and afternoon, respectively. Red arrows show those squares that their discretized direction is different from that in the morning pattern **c**. The gray lines in the map indicate railways.

In order to observe collective human flow patterns we first divide the urban map into a square lattice of units of sizes $500\times 500\hbox { m}^2$ and calculate the mean velocity vector averaged over moving people of non-zero velocity at each square in a time interval of 30 min snapshots as schematically shown in Fig. 1a-left. The time intervals of 30 min is applied from 5:00 to 25:00 ($=1$ a.m. in the next day). Then, for each square we calculate the projected component values of the mean velocity vector in the 4 directions, {north, east, south, west} and choose the direction with the largest component as the discretized representative direction of the square as shown in Fig. 1a-right. Figure 1c,d show examples of detailed flow pattern maps which are located at the small west part in the center of Tokyo (the purple square in the wider map, Fig. 1b), in the morning (7:30–8:00) and afternoon (13:30–14:00) of a weekday, respectively. In Fig. 1c a typical morning rush-hour flow pattern is observed. We can see many arrows directing to the right or bottom toward the center of Tokyo which is located at the right-down corner of the map. The arrows of the squares which include railways can be also seen to be highly correlated with pointing towards the city center. In Fig. 1d a typical afternoon flow pattern is shown with red arrows that indicate the flow directions that are different from the morning pattern seen in Fig. 1c. It suggests that the directions of arrows at afternoon do not point to a certain data and are more like random.

### Drainage basin structures and the population of moving people

**Figure 2:**
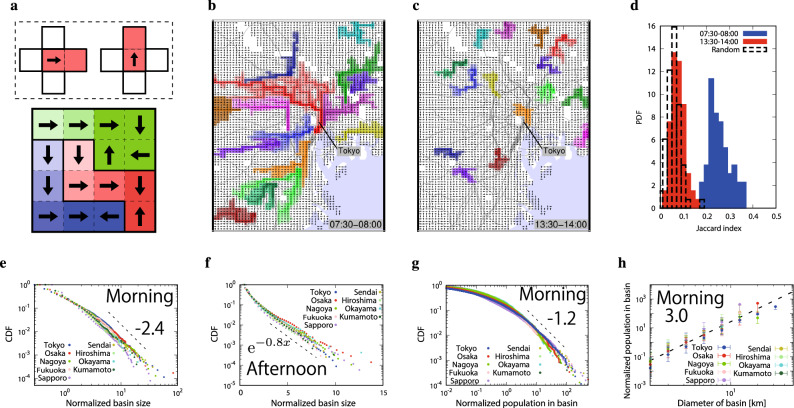
Drainage basins around Tokyo metropolitan area and basin size distributions for 9 cities. (**a**) The definition of uniquely identifying drainage basins. Each square with an arrow is regarded to be connected and to belong to the same drainage basin as the neighboring square in the direction of arrow. The set of connected squares defines a drainage basin. In the bottom figure there are 3 drainage basins, red, blue and green with color strength proportional to the number of upstream squares. (**b**,**c**) Flow maps of basin sizes in (**b**) the morning commuter rush hour (07:30–08:00) and (**c**) the afternoon (13:30–14:00) around Tokyo metropolitan area, where the largest 15 basins are shown in different color codes. The displayed area is the green area shown in Fig. 1b. The gray lines in the map represent the railways. (**d**) The Jaccard indexes representing overlap of the top 15 basins within the green line in Fig. 1b are calculated for all pairs of months, for the morning rush hour (blue), the afternoon (red), and randomly shuffled patterns (dotted lines). (**e**,**f**) Cumulative distribution functions of basin sizes for the morning rush hour and the afternoon for the 9 analyzed cities, respectively, where basin sizes are normalized by the mean basin size. The nine cities are the metropolitan regions of Tokyo, Osaka, Nagoya, Fukuoka, Sapporo, Sendai, Hiroshima, Okayama and Kumamoto. In the case of morning commuter rush hour, the CDFs are well approximated by a power law with an exponent close to −2.4 (the dashed line). In the afternoon, the CDFs are roughly approximated by an exponential function. (**g**) Cumulative distribution functions of number of moving people in basins for the morning rush hour, which follows a power law with the exponent close to −1.2, where the number of people are normalized by the mean number of moving people in all basins in the corresponding city. (**h**) The relation between the population of moving people in each basin and the diameter of its basin in log-log scale. The dotted line shows the scaling relation with an exponent very close to 3.0.

For characterizing the flow patterns quantitatively we introduce and develop the concept of drainage basin which has been useful in the study of river flow patterns^34^. We define basins for a discretized flow pattern in the following way. As shown in the upper part of Fig. 2a, we consider simply that the people in a square flow mainly into the neighboring square in the direction of the arrow, and we regard these two squares belonging to the same drainage basin. By applying this rule to all squares we can uniquely define drainage basin clusters as schematically shown in the lower part of Fig. 2a. The clusters are shown in different colors where the darkness is proportional to the number of upstream squares, implying that more people move in the darker areas. In Fig. 2b,c, the top 15 basins of Tokyo area are shown for the morning rush hour and the afternoon (see Fig. S5 in Supplementary Information for evening flow patterns). In the morning flow pattern (Fig. 2b), we can see several huge drainage basins pointing towards the center of Tokyo. In the afternoon pattern (Fig. 2c), the sizes of the basins are significantly smaller and there seems to be no specific flow direction.

We now consider the large drainage basin areas and test their similarity in different months. We first prepared 12 monthly averaged flow patterns for the year of 2015 in the morning and the afternoon just like Fig. 2b,c. Next we calculate the Jaccard index which is defined as the ratio of the overlapped area divided by the area of union, where the area is the top 15 drainage basins for each monthly flow pattern. Figure 2d shows that the values of Jaccard indexes between the morning basin patterns (blue) are much larger than those between the afternoon basin patterns (red) implying that the morning basin patterns are similar throughout the year, while the afternoon basin patterns are changing monthly. We also apply the same analysis to artificially made flow patterns in which the arrows are randomly shuffled (dashed line), and find that the values are close to the results of the afternoon. This random nature of the afternoon patterns is more directly confirmed by observing the mean velocity correlation between squares at distance *r* which decays at small *r* to 0 (see Supplementary Information, Fig. S4). We apply the above analysis to other 8 large cities in Japan, and confirm that the above properties are very similar.

Next we study the basin size distribution for each of the 9 largest cities for morning rush hours, see Fig. 2e. Here, the y-axis shows the cumulative distribution, that is, the probability that a randomly chosen basin’s size is larger than the value at the x-axis, which is normalized by the mean basin size of each distribution. We find that the distributions are well approximated by power laws based on applying the Kolmogorov-Smirnov test with the best estimated exponent values around $2.4 \pm 0.2$ (see the “Materials and methods”)^35^. The basin size distributions for the afternoon are shown in Fig. 2f in semi-log plot. We find that the basin size distributions in the afternoon are nearly linear for all cities implying that the size distributions can be roughly approximated by exponential functions. The basin size distributions for randomly shuffled flow patterns also follow exponential-like distributions of a similar slope.

**Figure 3:**
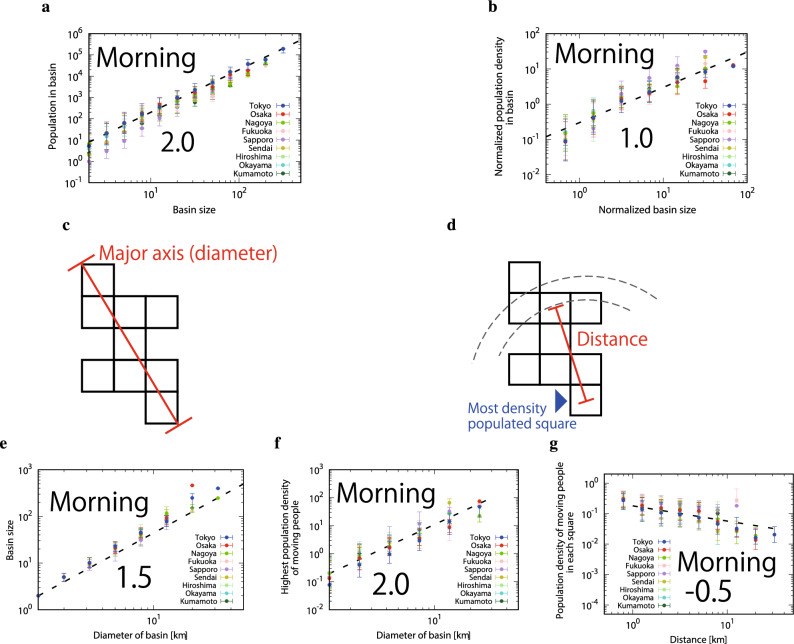
The fractal structure of drainage basin. (**a**) The number of moving people in drainage is proportional to the square of the drainage size. (**b**) The relation between the population density of moving people in basins and basin size with scaling exponent 1.0. (**c**) We define the major axis as diameter. (**d**) The distance is defined by the difference from the most densely populated square. (**e**) The plots show relation between basin area size $S_{b}$ and the major axis (diameter) $L_{b}$ with scaling exponent 1.5. **f** The maximum value of the population density of moving people in a basin of diameter $L_{b}$ with the scaling exponent is 2.0. (**g**) The population density in a basin scale with the distance from the most densely populated square as $r^{-0.5}$.

Next, we focus on the population of moving people in each drainage basin in the morning rush hour. In Fig. 2g, we find the CDFs are approximated by power laws by applying the Kolmogorov-Smirnov test with the best estimated exponent values around $1.2 \pm 0.2$ (see the “Materials and methods”)^35^. This difference between the power law exponents of the distributions for drainage basin area *S*, and the population of moving people *p* is surprising, since it means that population is not proportional to the drainage area. We suggest here that indeed these two quantities fulfill a non-trivial nonlinear relation as:

1 $$\begin{aligned} p \propto S^{2}, \end{aligned}$$

which is supported by Fig. 3a. Note that the formula has an error of about $\pm 0.2$ by error propagation of the power law exponents of the distributions for drainage basin area and the population of moving people. To further test this surprising nonlinear scaling we plot the population of moving people in each basin as a function of the basin diameter *L*, which is defined as the maximum distance between two points in the basin (see Fig. 3c), in Fig. 2h finding a novel cubic law as:

2 $$\begin{aligned} p \propto L^{3}, \end{aligned}$$

which contradicts the natural intuition of $p \propto L^{2}$. This result suggests that human flow in urban cities is not simply gathering people uniformly in the drain like the case of water flow, but the flow intensity is enhanced in an extra dimension ($L^{3}$, 3 dimensions) as basin increases causing extremely high density. From Eqs. (1) and (2) another non-trivial scaling relation is expected,

3 $$\begin{aligned} S \propto L^{1.5}, \end{aligned}$$

which means that the geometry of the basins, i.e., the area of main traffic, are characterized by a fractal structure with the dimension 1.5.

### The fractal structure of drainage basin

Figure 2 suggests that the population of moving people in a drainage is proportional to the square of the basin size (Eq. 1), and that drainage basin has a fractal structure (Eq. 3). We directly support these relations in Fig. 3a showing the relation between the population in a basin and the basin size. Also, the number of moving people is given by:

4 $$\begin{aligned} p_{b} \propto S_{b}^{2.0} \propto {\overline{\rho }}_{b}(S_{b})S_{b}, \end{aligned}$$

where ${\overline{\rho }}_{b}(S_{b})$ is the averaged population density of moving people in the b-th basin of size $S_{b}$. Here, the density ${\overline{\rho }}_{b}(S_{b})$ is defined as the total number of moving people in the basin $p_{b}(S_{b})$ divided by its area size $S_{b}$. As seen in Fig. 3b, ${\overline{\rho }}_{b}(S_{b})$ and $S_{b}$ have a linear relation. To better understand this unexpected scaling relation, we define a diameter (see Fig. 3c) and distance (see Fig. 3d) for each basin. The non-trivial 3-dimensional relation, Eq. (2), is equivalent to the following relation for each b-th basin:

5 $$\begin{aligned} p_{b}(L_{b}) \propto L_{b}^{3.0}, \end{aligned}$$

where $L_{b}$ is the diameter of the b-th basin. In Fig. 3e, the basin size is found to scale with a power 1.5 of the diameter $L_{b}$. Thus, we identify a new scaling relation:

6 $$\begin{aligned} S_{b}(L_{b}) \propto L_{b}^{1.5}. \end{aligned}$$

This implies that the fractal dimension of drains is $D=1.5$. Since, the population density is proportional to its drainage size, it suggests that the population density in basins is given by:

7 $$\begin{aligned} {\overline{\rho }}_{b}(L_{b}) \propto L_{b}^{1.5}, \end{aligned}$$

which is derived from $p_{b}(L_{b})$ divided by $S_{b}$.

To deeper understand the above finding, we assume that the population density of moving people in a basin is characterized by the distance from the most dense populated square, $\rho _{b}(r)$, where the distance *r* denotes the distance from the most dense square (which we call the center of drainage). The total number of moving people in a basin $p_{b}(S_{b})$ is then given as:

8 $$\begin{aligned} p_{b} = \int ^{L_{b}}_{1} \rho _{b}(r) \Delta S_{b}(r)dr, \end{aligned}$$

where $\Delta S_{b}(r)dr$ is the area of drainage basin at the distance *r* from the center between *r* and *rdr*, so that the area $S_{b}$ is given as:

9 $$\begin{aligned} S_{b}=\int ^{L_{b}}_{1} \Delta S_{b}(r)dr. \end{aligned}$$

From the fractal property, $S_{b}(L_{b}) \propto L_{b}^{1.5}$, we expect $\Delta S_{b}(r) \propto r^{0.5}$. We also assume the following power law functional form for the population density of moving people:

10 $$\begin{aligned} \rho _{b}(r) = \rho _{b,max}(L_{b})r^{-\alpha }, \end{aligned}$$

where $\rho _{b,max}(L_{b})$ is the maximum value of the population density of moving people in a drainage of diameter $L_{b}$. In Fig. 3f, we find that $\rho _{b,max}(L_{b})$ follows the following power law:

11 $$\begin{aligned} \rho _{b,max}(L_{b}) \propto L_{b}^{2.0}. \end{aligned}$$

Fig. 3g indicates the population density of moving people decreases with the distance from most densely populated square in each basin as $\rho _{b}(r) \propto r^{-0.5}$. Therefore, the population density of moving people is given as:

12 $$\begin{aligned} \rho _{b}(L_{b},r) = \rho _{b,max}(L_{b})r^{-\alpha }=L_{b}^{2.0}r^{-0.5}. \end{aligned}$$

Finally, the population of moving people in a basin is calculated as:

13 $$\begin{aligned} p_{b}(S_{b})= & {} \int ^{L_{b}}_{1} \rho _{b}(r) \Delta S_{b}(r)dr \nonumber \\\propto\, & {} \int ^{L_{b}}_{1} L_{b}^{2.0} r^{-0.5} r^{0.5} dr \nonumber \\\propto\, & {} L_{b}^{2.0} L_{b}^{1.0} \nonumber \\\propto\, & {} L_{b}^{3.0} \nonumber \\\propto\, & {} S_{b}^{2.0}. \end{aligned}$$

### Other scaling relations characterizing the city traffic

**Figure 4:**
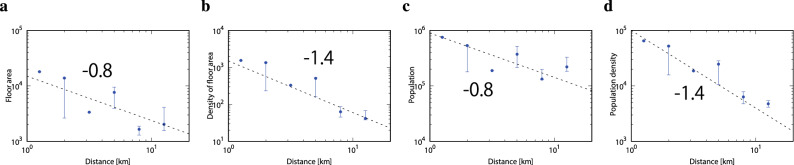
The relations between the daytime population and office floor area in Tokyo. (**a**) The office floor area is inversely proportional to $-0.8\pm {0.3}$ power of the distance from the center, i.e., the Imperial Palace. (**b**) The scaling relation between the density of the floor area in the city and the distance from Imperial Palace with the scaling exponent $-1.4\pm {0.3}$. (**c**) The daytime population is inversely proportional to $-0.8\pm {0.3}$ power of the distance from the Imperial Palace. (**d**) The scaling relation between the density of daytime population in the city and the distance from Imperial Palace with the scaling exponent $-1.4\pm {0.3}$.

To further and independently understand and support these unexpected scaling relations we analyze the office floor area and daytime worker population using the two governmental official census data^36,37^, which include the worker population and the office floor area for the 23 wards of Tokyo. We regard the Imperial Palace as the city center of Tokyo and the distance *r* for each ward is defined by the linear distance to the ward office. In Fig. 4a, we show the relation between floor area as a function of distance from the city center to quantify the effect of skyscrapers. The gross floor area of offices and shops in the ward is found to be inversely proportional to the $0.8\pm {0.3}$ power of the distance *r*. Also, in Fig. 4b, the density of the floor area in the ward $f_{A}$, which is defined by the gross floor area divided by the whole area of the ward, decreases proportional to the $-1.4\pm {0.3}$ power of the distance *r*. Next, we show in Fig. 4c,d that the daytime worker population in the city, *P*, and the daytime worker population density in the city, $\rho _{A}$, which is defined by the daytime worker population divided by the whole area of the ward, show quite similar scaling relations as the office floor area and the density of floor area in the ward, respectively. That is, the office floor area per person $\rho _{F}$ is roughly constant for any ward as naturally expected.

As seen from these results the city structure in view of human capacity is quite non-uniform, and the density of working people tends to increase towards the city center. These relations are the cumulative amount for the whole city which can be regarded as a potential driving force of the strong human flow from suburbs to the city center. The typical non-trivial scaling relation, Eq. (2), is a snapshot property of the resulting strong non-uniform human flow observed in each drainage basin at the most congested morning rush hour, see also further discussion in the “Materials and methods” Section, Eqs. (4) to (13) and Fig. 3.

## Discussion

In this paper we analyzed GPS data of location and velocity of over 2 hundred thousand users in Japan. As shown in Supplementary Information 1.1 and 1.2 individual user’s location changes have been observed from early morning to midnight using this data. Here, we did not pay attention to such individual traces, but we focused on collective motion of people around big cities. In order to characterize macroscopic human flow pattern we introduced a coarse-graining method described in “Velocity discretization” explained in detail in the “Materials and methods” section, and we naturally defined drainage basins as schematically shown in Fig. 2a. This velocity discretization procedure is a rough simplification, however, we believe that characteristics of macroscopic flow patterns are captured with this method.

The basin area distributions in the afternoon can be approximated by exponential functions, which is consistent with the assumption that during afternoon most of the moving directions are uncorrelated indicating that people move independently. On the other hand, in the morning rush hour there appear strong human flows toward the city center causing huge drainage basins. The cumulative basin size distributions at the peak rush hour are approximated by a power law with a non-trivial exponent about 2.4, which are the same for 9 big cities.

Relating to this morning rush hour property, we found additional scaling relations characterized by power laws such as the non-trivial three dimensional relation, Eq. (2), that is, the population of moving people in a drainage basin of diameter *L* is proportional to $L^{3}$. As discussed in the section, “The fractal structure of drainage basins”, this cubic law is shown to be consistent with the fractal geometry of basin structure with the fractal dimension 1.5 (Fig. 3e), and the power law decay of population density in each basin, $r^{-0.5}$ (Fig. 3g).

This power law decay of population density is expected to be deeply related to the structure of cities; skyscrapers with huge human capacity, that are located near the city center and many trains are gathered also towards the city center. Figure 4 shows examples of non-trivial scaling relations for daytime population and office floor area as a function of the distance from the city center confirmed for Tokyo. We expect that similar relations hold for any city.

We conjecture that our new view of macroscopic human flow patterns in metropolitan areas is applicable to all cities around the globe and reveals universal flow patterns within urban areas.

## Materials and methods

### The data

Our GPS data is provided by the Japanese private company, Agoop, which operates application programs of smart phones. The GPS data consists of the user ID, date, time, longitude, latitude, velocity in longitude and velocity in latitude, where velocities are estimated by Doppler effect of the electromagnetic wave frequency. The number of users is about 260,000 in Japan, and for each user the GPS data is collected every day except from 1 a.m. to 5 a.m. with intervals about 30 min. For protection of privacy, the user IDs are randomized every day. The observation period is the whole year of 2015. The total data size is about 1 TB. We applied the data trimming to the original data set in advance (see Fig. S3 in Supplementary Information).

### Velocity discretization

**Figure 5:**
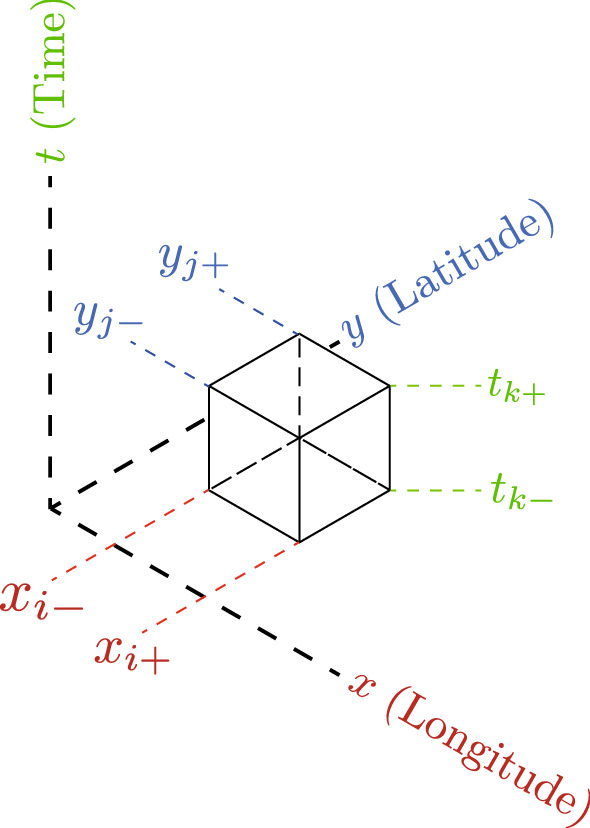
Labeling the location and time. In order to uniquely specify the location and time, we introduce a set of integers, *i*, *j*, *k*, for longitude, latitude, and time. The size of the squares are 500m by 500m, and the time interval is 30min. Data belonging to the space-time $A_{i,j,k} \equiv (x,y,t)\in [x_{i-},x_{i+}) \times [y_{j-},y_{j+}) \times [t_{k-},t_{k+})$ is represented by subscripts *i*, *j*, *k* when distinguishing both edges with plus or minus subscripts.

We divide the map into square lattice of sizes $500 \times 500 \,{\hbox {m}^2}$ (based on Japanese Industrial Standards) and calculate the mean velocity in each square by taking average of velocities of individuals with non-zero speed within the square in a time interval of 30 min. Since the update time intervals of the GPS position data is not constant but depends on users, we introduce a weighted average for the case that signals are transmitted for more than 2 times in an interval of 30 min. First, using data identified based on User’s IDs and time, we calculated $n_{{ID,T,k}}$, which is the transmission frequency of a user with ID number, *ID*, during the k-th time interval of 30 min on date *T*. We define $n_{{ID,T,i,j,k}}$, as the transmission frequency in the square which is located in the i-th in east-west direction and the j-th in south-north direction (see Fig. 5).

Existence probability that a user with a given ID exists in the square located at (*i*, *j*) during the time interval of k on the T-th day is defined as:

14 $$\begin{aligned} w_{{ID,T,i,j,k}}=\dfrac{n_{{ID,T,i,j,k}}}{n_{{ID,T,k}}}. \end{aligned}$$

Also, population in a square (*i*, *j*) at the k-th time interval is given as:

15 $$\begin{aligned} {p}_{{T,i,j,k}}= & {} \sum _{ID}{w_{{ID,T,i,j,k}}}, \end{aligned}$$

where the summation is taken over all user IDs. Next, we define each ID’ s average velocity in the square (*i*, *j*) of the k-th time intervals:

16 $$\begin{aligned} \overline{\varvec{v}}_{{ID,T,i,j,k}}=\dfrac{\sum _{(i,j,k)\ \in A_{i,j,k}}\varvec{v}_{{ID,t}}}{n_{{ID,T,i,j,k}}}, \end{aligned}$$

where $\varvec{v}_{{ID,t}}$ is the value uniquely determined from ID and time *t*, and we do not define this value in the case that the denominator is zero. The averaged velocity of the square (*i*, *j*) in the time interval *k* is defined as follows:

17 $$\begin{aligned} \overline{\varvec{v}}_{{T,i,j,k}}= & {} \dfrac{\sum _{ID} w_{{ID,T,i,j,k}}\overline{\varvec{v}}_{{ID,T,i,j,k}} }{{p}_{{T,i,j,k}}}\,\,\,\,if\,\,\,\Bigl ({p}_{{T,i,j,k}}\ne 0\Bigr ), \nonumber \\= & {} No\,Data\,\,\,\,if\,\,\,\Bigl ({p}_{{T,i,j,k}}=0\Bigr ). \end{aligned}$$

### Kolmogorov–Smirnov (KS) test for the power law distribution hypothesis

We conduct a statistical test according to the following procedure^20,35^.

1. We define the null hypothesis $\hbox {H}_{0}$ as the data fit with a power law and the alternative hypothesis $\hbox {H}_{1}$ as the data does not fit a power law.
2. The power law exponent in the following equation is estimated by the maximum likelihood estimation method. The estimated probability density function *f*(*x*) is
  18 $$\begin{aligned} f(x)=Cx^{-(\alpha +1)},\,\,\, x\in [x_{min},\infty ), \end{aligned}$$
  where the normalization constant is $C=\dfrac{\alpha }{x_{min}^{-\alpha }}$. The log-likelihood function is defined as:
  19 $$\begin{aligned} \ln {L(\alpha )}=n\ln {\alpha }-n\ln {x_{min}}-(\alpha +1)\sum _{i}^{n} \ln {\frac{x_{i}}{x_{min}}}, \end{aligned}$$
  where *n* indicates the number of data values used for the maximum likelihood estimation method. Differentiating the above equation by $\alpha$, the estimated $\alpha$ is given by:
  20 $$\begin{aligned} \alpha =n[\sum _{i}^{n}\ln {\frac{x_{i}}{x_{min}}}]^{-1}. \end{aligned}$$
  To estimate the distribution, $x_{min}$ must also be determined. The difference between the data and the estimated distribution is given by:
  21 $$\begin{aligned} D_{x_{min}}=sup|F_{data}(x)-F_{model}(x)|, \end{aligned}$$
  where $F_{data}(x)$ and $F_{model}(x)$ are the cumulative distribution function of the real data ($x\in [x_{min},\infty$) and the estimated model (exponent $\alpha$). The parameter $x_{min}$ that minimizes the value of $D_{x_{min}}$ is the optimal one.
3. The KS statistic *D* is defined as:
  22 $$\begin{aligned} D=sup|F_{data}(x)-F_{model}(x)|. \end{aligned}$$
  Taking the difference between the model and the data at each value of *x*, the maximum value is defined as *D*.
4. Next, 10000 random number data sets composed of n number of data obeying to the power law of the exponent $\alpha$ are created. KS statistic $D^{*}$ for each random number data set $F_{syn}(x)$ is given as:
  23 $$\begin{aligned} D*=sup|F_{syn}(x)-F_{model}(x)|. \end{aligned}$$
  We count the number of random samples which fulfill $D<D^{*}$, and the p-value is defined by dividing this number by the total number of random samples. As summarized in Tables 1 and 2, the basin size distributions and distributions of moving people in basins, can be regarded as power laws for all 9 cities.

**Table 1:** The test condition of 9 cities for basin size.

City	$\alpha$	$x_{min}$	p-value
Tokyo	2.57	5.95	0.9313
Osaka	2.10	3.99	0.8462
Nagoya	2.35	3.98	0.5891
Fukuoka	2.87	6.63	0.9984
Sapporo	2.36	2.08	0.0513
Sendai	2.44	2.96	0.9019
Hiroshima	2.26	2.06	0.7207
Okayama	2.48	2.72	0.5948
Kumamoto	2.46	2.72	0.7359

The mean values and standard deviations of the power law exponents is $-2.4\pm 0.2.$

**Table 2:** The test condition of 9 cities for moving people in basins.

City	$\alpha$	$x_{min}$	p-value
Tokyo	1.17	4.75	0.8334
Osaka	1.16	3.26	0.3329
Nagoya	1.69	7.86	0.9767
Fukuoka	1.24	2.51	0.9805
Sapporo	0.93	1.96	0.3687
Sendai	1.23	2.81	0.9994
Hiroshima	1.24	2.08	0.9535
Okayama	1.07	1.32	0.2364
Kumamoto	1.05	1.39	0.7708

The mean values and standard deviations of the power law exponents is $-1.2\pm 0.2.$

## Supplementary information

Supplementary Information.

## Data Availability

Our data cannot be open to public, but the same data can be purchased from a Japanese private company, Agoop, which sells “The location information big data which acquired from the smart phone app.”
