# Supplementary material for: Universal scaling laws of collective human flow patterns in urban regions
Source: Sci Rep. 2020 Dec 8;10:21405. doi: 10.1038/s41598-020-77163-2 (PMC7722863; doi:10.1038/s41598-020-77163-2)
Supplement: Supplementary file 1 — Supplementary Information. [file 41598_2020_77163_MOESM1_ESM.pdf]

Universal Scaling Laws of Collective Human Flow Patterns in Urban Regions Supplementary Material

Yohei Shida, Hideki Takayasu, Shlomo Havlin, Misako Takayasu

Contents

1 Basic properties of the mobile phone GPS data **2**

1.1 Returners and Travelers 2

1.2 Displacement distribution 2

1.3 Data trimming 3

1.4 Correlation of the averaged velocity as a function of distance 4

1.5 Evening flow patterns around Tokyo and size distributions 4

1.6 Drainage basins around Osaka and Nagoya metropolitan area 6

1.7 Drainage basins around Tokyo metropolitan area on holidays 6

1 Basic properties of the mobile phone GPS data

1.1 Returners and Travelers

To show that the data is reliable, we measure the displacement by each person on weekdays and compare with previous results [1–4]. We first introduce categorization of users into 2 groups; returners whose location in the morning is the same as at midnight, and travelers whose locations are different in the morning and at midnight. Users that send data 30 times or more per day and the difference between their home and the last stop is within 100 m are regarded as returners, and 10 km or more are regarded as travelers. Users who do not belong to either of these cases are excluded from our analysis.

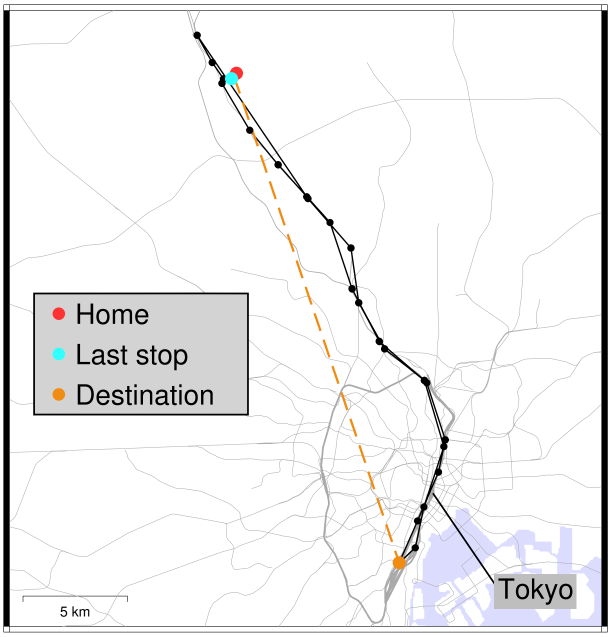

Figure S1: An example of a returner trajectory. The red dot (home) is the earliest point sent before 7 o'clock. The blue dot (last stop) represents the last point, which is the latest sent point after 21:00. The orange point shows the point farthest from the home, and the travel distance of the orange dashed line is defined as a straight line.

1.2 Displacement distribution

We analyzed the distributions of travel distances of individuals in Fig. S2a. We categorize the users into 2 types, returners whose location at 5 a.m. is the same as that at 12 p.m. midnight, and travelers whose locations are different in the morning and in the midnight. The faction of returners is about 88%. In Fig. S2a "Total" means the summation of distances of location changes in one day, and the "Straight line" means the maximum distance between the location at 5 a.m. morning (home) and the farthest place from home in the same day. We find that there is a long tail in the travel distance distributions of returners which can be approximated by a power law with an exponent about -1.5, and Fig. S2b indicates that the total travel distance is about 2.8 times of the straight line distance because any travel route has some zig-zag. As seen in Fig. S2a the distance distributions of travelers are very different from those of returners, and the distributions are approximated by a truncated power law with the exponent -0.75 and the cut-off scale of 500 km. These results are consistent with previous studies about individual travel distances [1–4].

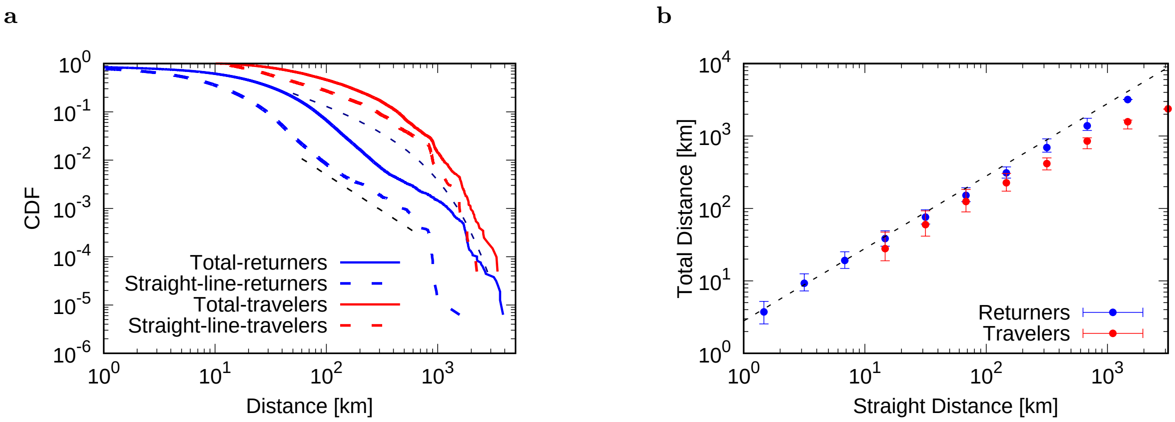

Figure S2: Displacement distribution. **a**, Log-log plot of CDF (cumulative distribution function) of traveled distances in a day. The blue line shows the CDF of the total sum of distances for returners. The dashed blue line shows CDF of the straight distances between home and the farthest place from home in a day. The gray dashed straight line shows a power law distribution with the exponent -1.5. The red line shows the CDF of the total sum of traveled distances for travelers, and the red dashed line shows the CDF of the straight-line distance for travelers. The gray dashed curved line shows a truncated power law with an exponent -0.75 with a cut off at 500 km. **b**, Relationship between the straight-line distance and the total sum of traveled distances. The blue dots for returners follow a linear relation approximated by a dashed black line, showing that the total distance is 2.8 times the straight line distance.

1.3 Data trimming

We describe the data trimming which we applied to the original data set. Fig. S3 shows all cities probability density function of the speed, which is defined by the square root of the sum of squares of velocity components in longitude and latitude, in semi-log plot. It is seen that the density decreases sharply around 320 km/h, which agrees with the maximum speed of trains in Japan, and we remove those data larger than this value as abnormal values (namely, we neglect travelers by airplanes). Based on the governmental information [5], data observed on the uninhabitable area such as on the ocean, river or mountain were also categorized as abnormal and removed from the data [5].

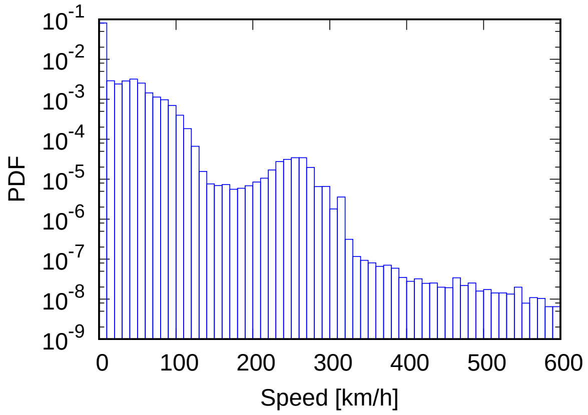

Figure S3: PDF(Probability Density Function) of speed. The speed, i.e., the square root of the sum of squared velocity components is calculated for all records in October 2015 in Japan. The small peak around 250 km/h corresponds to Shinkansen (the bullet Train).

1.4 Correlation of the averaged velocity as a function of distance

We calculate the correlation of velocities of squares in distance r km at the same time, $C(\boldsymbol{r})$, which is defined by the inner product of the averaged velocities as follows:

$$\begin{aligned} C(\boldsymbol{r}) &= \left\langle \frac{\widehat{\boldsymbol{v}}_{x,T,i,j,k}\widehat{\boldsymbol{v}}_{x,T,i',j',k} + \widehat{\boldsymbol{v}}_{y,T,i,j,k}\widehat{\boldsymbol{v}}_{y,T,i',j',k}}{\|\widehat{\boldsymbol{v}}_{T,i,j,k}\|\|\widehat{\boldsymbol{v}}_{T,i',j',k}\|} \right\rangle_{\boldsymbol{r}}, \\ \widehat{\boldsymbol{v}}_{x,T,i,j,k} &= \overline{\boldsymbol{v}}_{x,T,i,j,k} - < \overline{\boldsymbol{v}}_{x,T,k} >, \\ \widehat{\boldsymbol{v}}_{y,T,i,j,k} &= \overline{\boldsymbol{v}}_{y,T,i,j,k} - < \overline{\boldsymbol{v}}_{y,T,k} >, \end{aligned} \tag{1}$$

where $< \overline{\boldsymbol{v}}_{x,T,k} >$ is the mean value of $\overline{\boldsymbol{v}}_{x,T,i,j,k}$ averaged over all (i,j), $\|\widehat{\boldsymbol{v}}_{T,i,j,k}\|$ is the the norm, and $< >_r$ denotes an average taken over all (i,j,i',j') which fulfills $\sqrt{(i-i')^2+(j-j')^2} = r$. From Fig. S4 we find that the correlation is positive for distance less than 30 km in the morning, while the correlation vanishes except very short distance in the afternoon. From this follows that the velocity directions in the afternoon is nearly random.

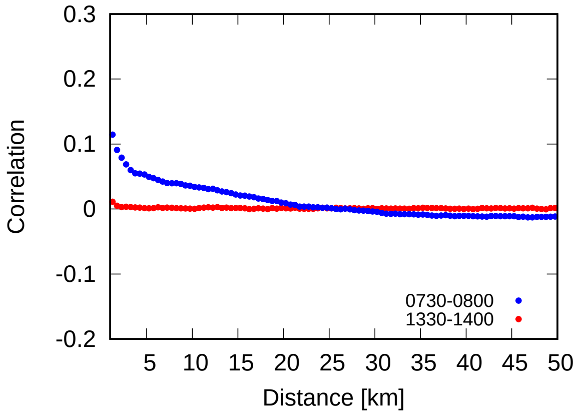

Figure S4: Correlation between velocities in squares at distance r. Blue dots are for the morning rush hour, and red dots are for the afternoon. In an artificial case the velocities are randomized, the correlation is 0 theoretically for any non zero distance.

1.5 Evening flow patterns around Tokyo and size distributions

We showed, in the main text, only morning and afternoon patterns. In addition, we show here, in Fig. S5a the evening flow pattern. As expected we can clearly see that many arrows are directed just the opposite compared with the morning pattern. In Fig. S5b, we show the top 15 basins of Tokyo area for the evening. In the evening, all large basins are directed towards outside the city center implying that people go home in suburban regions. The size distributions of basins for the evening are plotted in Fig. S5c. It is seen that the distributions are closer to the morning rush hour with limitation to the largest value, which can be well approximated by truncated power laws.

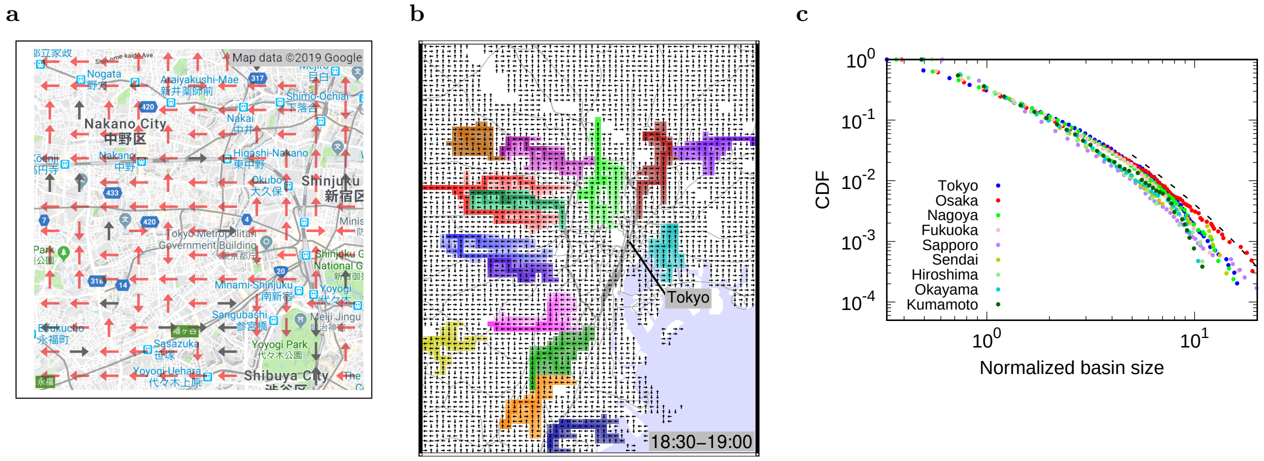

Figure S5: Evening flow patterns around Tokyo and the corresponding basin size distributions. **a**, Discretized flow patterns with the map in the evening (between 18:30-19:00). Red arrows indicate those squares where the discretized direction is different from the morning pattern. **b**, Flow map in the evening (18:30-19:00) around Tokyo metropolitan area, with the largest 15 basins colored by different colors. The gray lines in the map indicate the railroads. **c**, Cumulative Distribution Functions of basin sizes for the evening return rush hour, where each CDF is normalized by the mean cluster size. The nine metropolitan regions are Tokyo, Osaka, Nagoya, Fukuoka, Sapporo, Sendai, Hiroshima, Okayama and Kumamoto. The CDFs in the evening are well approximated by a truncated power law function proportional to $x^{-2}e^{\frac{-x}{10}}$ shown by the dashed curve. Note that the number 10 represents the typical size of clusters (number of squares) above which the power law stop to exist.

1.6 Drainage basins around Osaka and Nagoya metropolitan area

In addition to Tokyo metropolitan area, we show here, in Fig. S6, examples of flow pattern of Osaka and Nagoya metropolitan areas for the morning rush hour, afternoon, and evening.

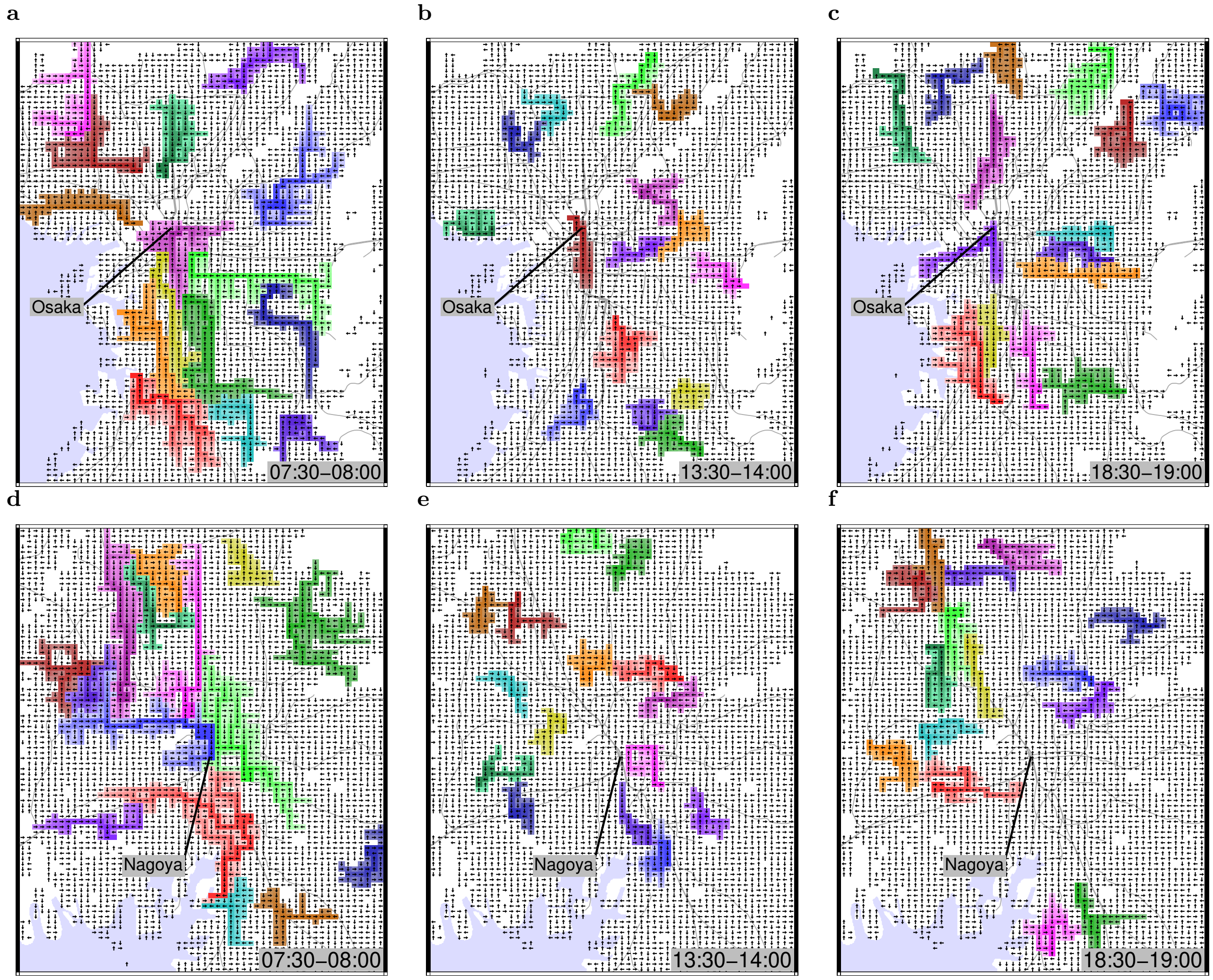

Figure S6: Drainage basins around Osaka and Nagoya metropolitan area. a-f, Flow maps in the morning commuter rush hour (07:30-08:00), the afternoon (13:30-14:00), and the evening (18:30-19:00) around Osaka (including Osaka, Kyoto, Hyogo, Nara) and Nagoya (including Aichi, Mie, Gifu, Shida) metropolitan area, respectively, with the largest 15 basins colored by different colors. The gray lines in the maps show the railroads.

1.7 Drainage basins around Tokyo metropolitan area on holidays

We now show in Fig. S7, the flow patterns on holidays and compare them with weekdays in Fig. 2 in the main text. At first glance, it looks that there is no difference between drainage basin patterns on holidays (shown in Figs. S7a, S7b and S7c) and weekdays, but the CDF shows a large difference. In Fig. S7d, we see in the distributions that the sizes of large drainage basins are much smaller than the case of weekdays, implying missing of commuter rush hour. On the other hand, due to the difference in lifestyle between holidays and weekdays, the human flow during the daytime on holidays clearly deviates from the distribution for random patterns indicating attractive flows to suburban cities.

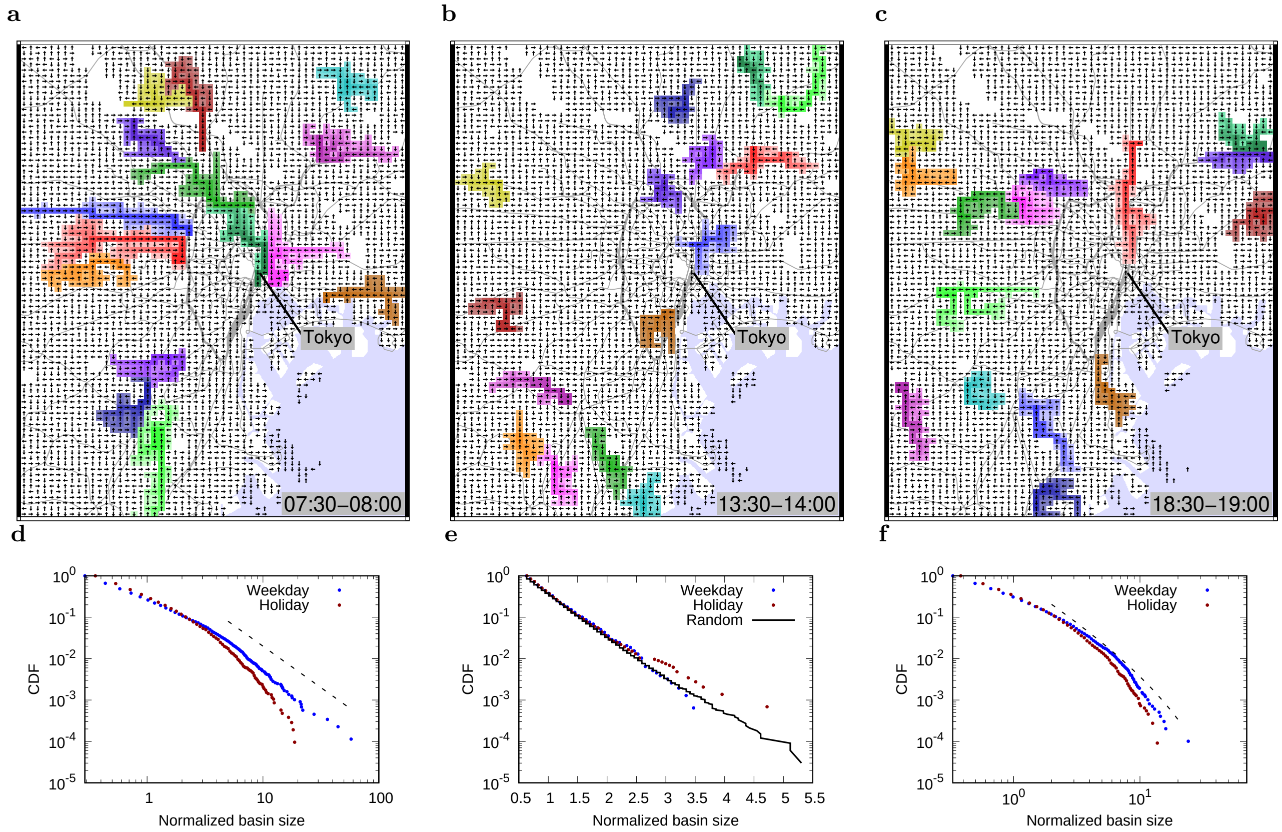

Figure S7: Drainage basins around Tokyo metropolitan area on holidays and basin area size distributions. a-c, Flow maps in the morning commuter rush hour (a. 07:30-08:00), the afternoon (b. 13:30-14:00), and the evening (c. 18:30-19:00) around Tokyo metropolitan area on holidays (Saturday, Sunday and public holidays), respectively, with the largest 15 basins colored by different color codes. The gray lines in the map are the railroads. **d-f**, Cumulative Distribution Functions (CDF) of basin sizes for (d) the morning rush hour, (e) the afternoon, and (f) the evening return rush (f), respectively, where the basin areas in x-axis are normalized by its averaged basin sizes. The CDFs of weekdays are plotted by blue lines for comparison, showing significantly larger basin sizes in morning and evening.

References

[1] Brockmann, D., Hufnagel, L. & Geisel, T. The scaling laws of human travel. *Nature* **439**, 462 (2006).

[2] González, M. C., Hidalgo, C. A. & Barabási, A.-L. Understanding individual human mobility patterns. *Nature* **453**, 779 (2008).

[3] Lu, X., Bengtsson, L. & Holme, P. Predictability of population displacement after the 2010 haiti earthquake. *Proceedings of the National Academy of Sciences* **109**, 11576–11581 (2012).

[4] Noulas, A., Scellato, S., Lambiotte, R., Pontil, M. & Mascolo, C. A tale of many cities: universal patterns in human urban mobility. *PloS one* **7**, e37027 (2012).

[5] National land numerical information download service. `http://nlftp.mlit.go.jp/ksj-e/index.html`. (Accessed on 09/12/2019).
